# Loss of expression of HDAC-recruiting methyl-CpG-binding domain proteins in human cancer

**DOI:** 10.1054/bjoc.2001.2041

**Published:** 2001-10

**Authors:** C Müller-Tidow, K Kügler, S Diederichs, S Klümpen, M Möller, U Vogt, R Metzger, P M Schneider, W E Berdel, H Serve

**Affiliations:** Department of Medicine, Hematology and Oncology, University of Münster, Germany; European Laboratory Association, Ibbenbüren, Germany; Department of Visceral and Vascular Surgery, University of Cologne, Germany

**Keywords:** CpG-methylation, *MeCP2*, *MBD2*, cancer

## Abstract

Dysregulation of CpG-methylation is a common feature of many human cancers and tumour suppressor genes can be silenced by hypermethylation. Recently, 2 methyl-CpG-binding domain proteins have been linked to gene inactivation by their ability to recruit co-repressors and HDAC-activity to methylated gene promoters. Here, we have analysed mRNA expression of these genes, *MeCP2* and *MBD2*, in a wide variety of primary human tumours. In solid tumours, expression levels of *MBD2* (57/71) and *MeCP2* (64/71) were significantly reduced in the majority of primary tumours as detected by quantitative real-time RT-PCR. Western blot analyses of MeCP2 in matched tumour–normal samples of patients with non-small-cell lung cancer (NSCLC) indicated reduced protein in a significant percentage of patients. In acute myelogenous leukaemia (*n* = 26), expression levels were only slightly reduced and did not differ between samples analysed at diagnosis or at the time of relapse. In early-stage NSCLC (*n* = 70) expression of MeCP2 and MBD2 was significantly lower in squamous cell carcinoma than in adenocarcinoma or large cell carcinoma (*P* = 0.03 and *P* = 0.01). To further elucidate the mechanisms of gene regulation, we analysed MeCP2 and MBD2 regulation during haematopoietic differentiation. No significant changes in MeCP2 or MBD2 expression were found when NB4 cells were differentiated toward granulocytes suggesting that neither differentiation nor cell cycle status were relevant for the reduced expression of these genes in human cancer. In conclusion, the significant loss of MeCP2 and MBD2 expression in human cancers suggests a potential role of this phenomenon in the development of solid human tumours. © 2001 Cancer Research Campaign  http://www.bjcancer.com


*British Journal of Cancer* (2001) **85**(8), 1168–1174
© 2001 Cancer Research Campaign
doi: 10.1054/ bjoc.2001.2041, available online at http://www.idealibrary.com on IDEAL
http://www.bjcancer.com

### Loss of expression of HDAC-recruiting methyl-CpG-binding domain proteins in human cancer

**C Müller-Tidow[1], K Kügler[1], S Diederichs[1], S Klümpen[1], M Möller, U Vogt[2], R Metzger[3], PM Schneider[3], WE Berdel[1] and H Serve[1]**

[1]Department of Medicine, Hematology and Oncology, University of Münster, Germany; [2]European Laboratory Association, Ibbenbüren, Germany; [3]Department of Visceral and Vascular Surgery, University of Cologne, Germany

**Summary** Dysregulation of CpG-methylation is a common feature of many human cancers and tumour suppressor genes can be silenced by hypermethylation. Recently, 2 methyl-CpG-binding domain proteins have been linked to gene inactivation by their ability to recruit co-repressors and HDAC-activity to methylated gene promoters. Here, we have analysed mRNA expression of these genes, *MeCP2* and *MBD2*, in a wide variety of primary human tumours. In solid tumours, expression levels of *MBD2* (57/71) and *MeCP2* (64/71) were significantly reduced in the majority of primary tumours as detected by quantitative real-time RT-PCR. Western blot analyses of MeCP2 in matched tumour–normal samples of patients with non-small-cell lung cancer (NSCLC) indicated reduced protein in a significant percentage of patients. In acute myelogenous leukaemia ($n$ = 26), expression levels were only slightly reduced and did not differ between samples analysed at diagnosis or at the time of relapse. In early-stage NSCLC ($n$ = 70) expression of MeCP2 and MBD2 was significantly lower in squamous cell carcinoma than in adenocarcinoma or large cell carcinoma ($P$ = 0.03 and $P$ = 0.01). To further elucidate the mechanisms of gene regulation, we analysed MeCP2 and MBD2 regulation during haematopoietic differentiation. No significant changes in MeCP2 or MBD2 expression were found when NB4 cells were differentiated toward granulocytes suggesting that neither differentiation nor cell cycle status were relevant for the reduced expression of these genes in human cancer. In conclusion, the significant loss of MeCP2 and MBD2 expression in human cancers suggests a potential role of this phenomenon in the development of solid human tumours. © 2001 Cancer Research Campaign http://www.bjcancer.com

**Keywords**: CpG-methylation; *MeCP2*; *MBD2*; cancer

Methylation at CpG dinucleotides is a common feature of the genomic organization of most higher organisms (Singal and Ginder, 1999; Walsh and Bestor, 1999).

The physiological pattern of methylation is established during embryonic development (Brandeis et al, 1993). Subsequently, methylation patterns are passed on to the daughter cells during mitosis. During the DNA replication process, DNMT1, a methyltransferase with a specificity for hemimethylated DNA, adds methyl groups to the newly synthesized DNA strand (Pradhan et al, 1997). The physiological role of CpG-methylation in embryogenesis and development has not been entirely defined, but the deletional mutant of DNMT1 proved to be embryonically lethal in mice (Li et al, 1992).

A role of methylation for tissue-specific regulation of gene expression has been discussed, but we and others have recently shown that the role of methylation in tissue–specific gene repression is limited (Warnecke and Clark, 1999; Müller et al, 2000a). While the role of CpG-methylation in normal development is unclear, an important role for CpG-methylation has been proposed in the pathogenesis of human cancers (Bird, 1996).

Changes in the patterns of CpG-methylation appear to be an intrinsic feature of human malignancy (Laird and Jaenisch, 1996). Modern technology such as restriction genomic landmark scanning has proven that significant changes in the genomic methylation pattern occur in probably all human tumours (Liang et al, 1998). The relevance of these phenomena and the mechanisms leading to the establishment of altered CpG-methylation are unknown. Besides changes in methylation in general, several tumour suppressor genes have been proposed to be silenced by aberrant methylation of their promoter region (Corn et al, 1999; Kawano et al, 1999). For example, the p16ink4A locus is deleted in most cell lines but is structurally not affected in the majority of primary tumours (Shapiro et al, 1995; Swafford et al, 1997). Expression of p16 is still lost in many primary tumours and hypermethylation which correlates well with gene silencing is proposed to be the relevant mechanism (Nakamura et al, 1999; Song et al, 2000).

However, the mechanisms of gene silencing by methylation are poorly understood. While the binding of some transcription factors appears to be inhibited by CpG-methylation at consensus-binding sites, others are not (Gaston and Fried, 1995; Singal and Ginder, 1999). Indirect mechanisms have been hypothesized to be relevant for gene silencing in vivo. The finding that methyl-CpG-binding domain (MBD) proteins can suppress transcription from methylated promoters suggests an attractive hypothesis for the silencing of methylated genes in cancer (Nan et al, 1997). The recruitment of histone deacetylase activity (HDAC) by members of the MBD protein family has linked their repressor activity to known transcriptional co-repressors that are associated with HDAC-recruitment (Jones et al, 1998). So far repressor activity has been confirmed for MBD1, MBD2 and MeCP2 (Nan et al, 1997; Ng et al, 1999, 2000). Two of these transcriptional repressors, MBD2 and MeCP2, can recruit co-repressors and HDACs to methylated DNA (Nan et al, 1998; Ng et al, 1999).

*Received 18 December 2000*
*Revised 30 May 2001*

*Correspondence to:* C Müller-Tidow

*MeCP2* is the so far best studied member of the MBD family and homozygous deletions of this gene are embryonically lethal (Tate et al, 1996). More evidence for the importance of MeCP2 derived from the recent finding that mutations of MeCP2 occur at a high frequency in patients with RETT syndrome which is characterized by a progressive neurodegenerative disorder (Amir et al, 1999). The potential role of MeCP2 in human cancers is currently unknown.

Even less is known about MBD2 which also suppresses transcription from methylated promoters. A recent report suggested that MBD2 expression is reduced in gastric and colon cancer but so far, there has been no systematic evaluation of its potential role in human cancer (Kanai et al, 1999).

In the current study, we systematically examined MeCP2 and MBD2 mRNA expression in a wide variety of human tumours. Several important findings were obtained: very low levels of MeCP2 and MBD2 could be detected in carcinoma samples derived from breast, colon, lung, and ovarian cancer. The loss of expression of MeCP2 or MBD2 did not have prognostic impact on survival for patients with early stage non-small-cell lung cancer.

On the other hand, MBD2 and MeCP2 expression levels in acute myeloid leukaemia were reduced by about 50%, indicating that tumour-type-specific differences in the degree of loss of expression occur.

Finally, loss of MBD2 or MeCP2 expression was not associated with the degree of differentiation or cell cycle distribution since granulocytic differentiation of NB4 leukaemic cells or monocyctic differentiation of U937 cells did not alter MBD2 or MeCP2 expression levels.

Taken together, our data show that the loss of MBD2 and MeCP2 expression is a common feature of a wide variety of human cancers.

### MATERIAL AND METHODS

### Tumour samples

Fresh tumours from patients with breast cancer ($n$ = 15), non-small-cell lung cancer (NSCLC) ($n$ = 14), cervical carcinoma ($n$ = 6), endometrium carcinoma ($n$ = 15), ovarian cancer ($n$ = 15), and colon cancer ($n$ = 6) were obtained at a molecular diagnostic laboratory after morphological dissection of the tumour by a pathologist.

Another group of patients with early stage NSCLC ($n$ = 70) has been described previously (Müller-Tidow et al, 2001). Briefly, tumour specimens were obtained at the time of initial surgery at a University hospital in Germany. Samples were snap frozen in liquid nitrogen and stored at –80˚C. Only samples from individuals with stages I to IIIA disease that were resected without pathological evidence for remaining tumour (RO resection) were included in this study. In addition, patients who survived for less than 90 days after surgery were excluded. Patients with stage IIIA tumours received radiation therapy after surgery. All patients were followed up for a minimum period of 5 years.

Bone marrow aspirates from patients with acute myelogenous leukaemia either at diagnosis ($n$ = 16) or at relapse ($n$ = 8) were obtained at the University of Münster for diagnostic reasons. These samples contained at least 70% blast cells by microscopic analysis. Samples from patients in complete remission ($n$ = 9) served as controls.

Control samples from a wide variety of organs were obtained from Clontech (Heidelberg, Germany). These samples contained cDNA pooled from 2 (brain and lung), 7 (ovary), 20 (colon), and up to 550 (leukocytes) individuals.

### RNA isolation and cDNA preparation

For RNA preparation, samples were disrupted into small pieces and RNA was isolated from tumour samples using Trizol reagent (Gibco, Life Technology) or RNeasy (Qiagen). A total of 1 µg RNA of each sample was reverse transcribed using an oligo-d(T) primer and RNase $H^-$ MMLV reverse transcriptase according to the protocol of the manufacturer (Promega). The cDNA was diluted to give a total volume of 200 µl, and 5 µl of this dilution was used for each PCR reaction. The quality of the cDNA was confirmed by amplification of glyceraldehyde-3-phosphate-dehydrogenase (GAPDH, cytosolic protein) or TATA-binding protein (TBP, nuclear protein) (see below) and only samples with consistent and strong amplification were included into the final analyses.

### Analyses of gene expression by real-time quantitative RT-PCR

The quantitation of mRNA levels was carried out using a real-time fluorescence detection method. The cDNA was prepared as described above and amplified by PCR in the ABI prism 7700 sequence detector (PE Biosystems, Foster City, CA). The primer and probe sequences used were published previously (Müller et al, 2000a). All primer and probe combinations were positioned to span an exon–exon junction. When genomic DNA was used as a template, no bands were seen after PCR amplification. The probes were labelled at the 5′ end with VIC (GAPDH probe) or with FAM (all others) and at the 3′ end with TAMRA which served as a quencher. The 5′ to 3′ nuclease activity of the Taq polymerase cleaved the probe and released the fluorescent dyes (VIC or FAM) which were detected by the laser detector of the sequence detector (Heid et al, 1996). After the detection threshold was reached, the fluorescence signal was proportional to the amount of PCR product generated. Initial template concentration can be calculated from the cycle number when the amount of PCR product passed a threshold set in the exponential phase of the PCR reaction. Relative gene expression levels were calculated using standard curves generated by serial dilutions of U937 cDNA. The relative amounts of gene expression were calculated by using the expression of GAPDH or TBP as an internal standard. At least 2 independent analyses were performed for each sample and each gene. Analyses of gene expression data were performed without knowledge of patient data.

### Western blot analysis

Protein was extracted from frozen tumours and corresponding normal lung tissue using radio-immunoprecipitation-assay buffer (RIPA) and sonication. Protein lysates were cleared by centrifugation and Western blotting was performed as previously described (Müller et al, 2000b). The primary anti-MeCP2 antbody was puchased from Upstate Biotechnology and was used at a 1:200 dilution. An anti-actin antibody was used to control for equal protein loading.

### Statistical analyses

Statistical data analyses were performed using SPSS 9.0 and 10.0 for Windows. When 2 groups with similar variance were compared, student's *t*-test was used. Otherwise, the Mann–Whitney

© 2001 Cancer Research Campaign

U-test was used. Means of several groups were compared with one-way analyses of variance (ANOVA). Kaplan–Meier plots were statistically evaluated by the log-rank test. All *P* values indicate 2-sided comparisons and a $P < 0.05$ was considered as significant.

### Differentiation of leukaemic cell lines

NB4 leukaemic cells were grown in RPMI supplemented with 10% fetal calf serum. For the induction of differentiation, cells were pulsed for 30 min with dimethylsulfoxide (DMSO) (Chih et al, 1997). Subsequently, all-trans retinoic acid (ATRA) ($10^{-6}$ M) was added and differentiation was followed by morphology. In addition, expression of a differentiation antigen, CD11b was analysed by flow cytometry using standard protocols. For cell cycle analyses, $10^6$ cells were harvested at the indicated time points and fixed in 70% ice-cold ethanol. DNA amount was quantitated by propidium iodine staining of nuclei and analyses were performed with a FACScan flow cytometer.

U937 cells were grown in RPMI supplemented with 10% fetal calf serum and differentiated with 12-O-tetradecanoylphorbol-13-acetate (TPA) (Santoli et al, 1983).

### RESULTS

### Expression of MeCP2 and MBD2 in healthy organs

We used quantitative real-time RT-PCR to analyse mRNA expression levels in a wide variety of human organs as well as in different human cancers. Probes were labelled with a fluorochrome at the 5′ end and with a quencher molecule at the 3′ end. In addition, probes were designed to span an exon–exon junction to avoid amplification of genomic DNA. Consequently, RT-minus controls and genomic DNA did not lead to an increase in signal intensity in the PCR reaction (data not shown). The average mRNA expression levels of the HDAC-recruiting methyl-CpG-binding domain proteins were then analysed in a wide variety of human organs by using quantitative real-time RT-PCR (Figure 1A).

Both genes were found to be expressed in all organs that we have analysed. Highest levels of MeCP2 and MBD2 mRNA were found in ovary and colon, respectively. Very low levels of MeCP2 were detected in testis, whereas expression of MBD2 was especially low in brain. Interestingly, expression levels varied up to 60-fold between different healthy tissues.

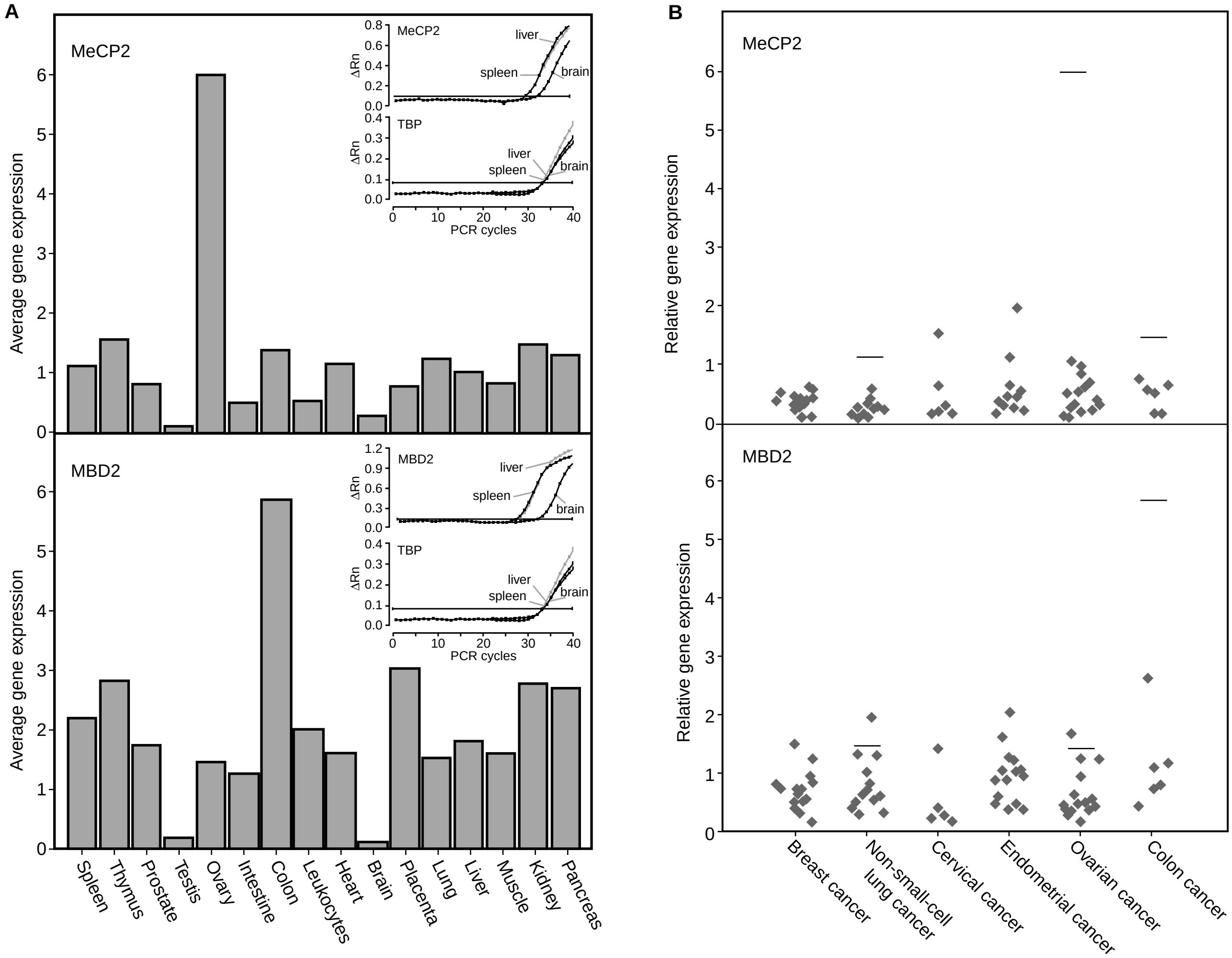

**Figure 1** Expression of MeCP2 and MBD2 mRNA in normal human organs and in a panel of primary human cancers. (**A**) Expression of MeCP2 and MBD2 in normal human organs was analysed in a panel of human normal cDNAs which were derived from pooled RNA from healthy individuals (see Material and Methods). Expression levels were standardized using TBP expression. (**B**) Expression levels of MeCP2 and MBD2 are shown for 71 primary human cancers from different locations. Average expression levels of pooled cDNA from healthy lung (from 2 individuals), ovary (from 7 individuals), and colon (from 20 individuals) are indicated by a dash. Expression levels were standardized by TBP expression

*© 2001 Cancer Research Campaign*

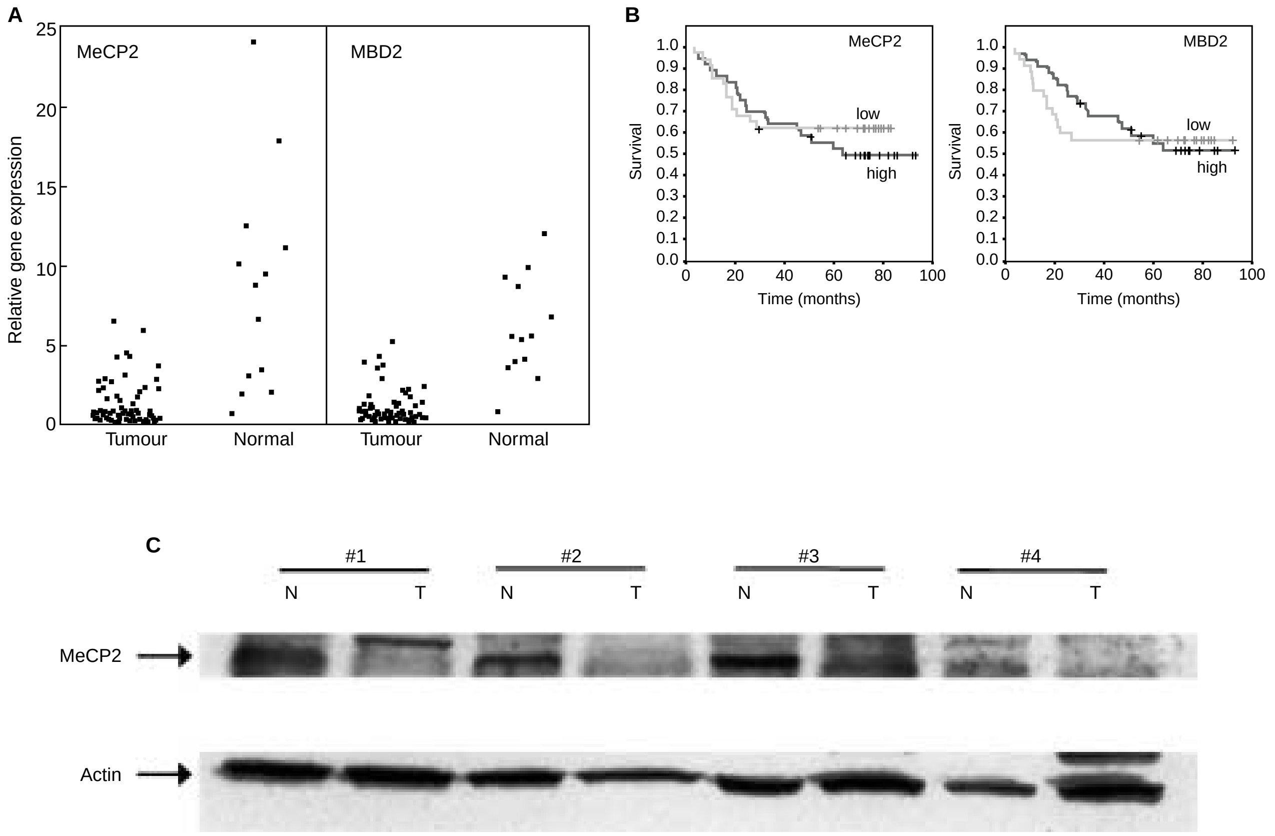

**Figure 2** Analyses of MeCP2 and MBD2 expression in non-small-cell lung cancer. (**A**) Expression of MBD2 and MeCP2 in a panel of early stage non-small-cell lung cancer ($n$ = 70) and in corresponding normal lung tissue specimens ($n$ = 12). Expression levels were standardized for GAPDH expression. Expression of MeCP2 ($P$ = 0.0001) and MBD2 ($P$ = 0.0001) was significantly lower in tumour samples than in controls. (**B**) Patients were divided in those with 'low' and 'high' MBD2 and MeCP2 expression according to the median of expression. Kaplan–Meier survival analyses are shown. No significant differences in survival were found for MBD2 or MeCP2 low vs high-expressing tumours. (**C**) 15 mg of protein lysates from matched tumour (T) and normal (N) samples from patients with NSCLC were analysed for MeCP2 protein expression by Western blot analysis. Thereafter, the blot was stained with anti-actin antibody to visualize equal protein loading. Most of the analysed patients expressed significantly reduced or no MeCP2 protein compared to normal lung tissue

### Expression of MeCP2 and MBD2 in solid cancers

Next, we focused on mRNA expression levels of MeCP2 and MBD2 in a panel of primary human carcinomas. Tumours were obtained for molecular diagnostic reasons and were microscopically dissected. Expression levels of MBD2 and MeCP2 in primary tumours derived from ovary, colon and lung were reduced by 20 to 80% compared to the mean levels reached in healthy controls. (Figure 1B). Low levels of expression were seen in breast cancer, higher expression levels in endometrium cancer. Interestingly, reduced expression similarly occurred for MeCP2 as well as for MBD2 resulting in a significant degree of linear correlation between these genes in this panel of primary tumours (r = + 0.4, $P$ = 0.01). These initial findings suggested a strong association between carcinomas and reduced MBD2 and MeCP2 expression.

To confirm these findings we used a previously well characterized group of patients with early stage (I to IIIA) non-small-cell lung cancer. 12 samples from disease-free lung tissue served as negative controls. In addition, a different housekeeping gene, *GAPDH*, was used for standardization. Again, expression levels of MeCP2 and MBD2 were much lower in tumour samples than in healthy control tissue (Figure 2A). Also, we detected that the expression of MBD2 and MeCP2 was neither associated with the stage of the disease, the sex of the patient nor with the histological grading (data not shown). In addition, MBD2 and MeCP2 were not associated with the subsequent development of metastasis or the patients' overall survival (Figure 2B).

To analyse whether mRNA expression levels corresponded to protein expression, Western blot analyses for MeCP2 were performed (Figure 2C). Protein lysates from matched tumour–normal samples were prepared for 9 patients with NSCLC. Western blotting analyses demonstrated that MeCP2 protein expression was significantly reduced in 5/9 NSCLC tumours compared to matched normal lung tissue from the same patient. These data showed evidence that at least MeCP2 expression in NSCLC was not only reduced on the mRNA level but also on the protein level.

### MBD2 and MeCP2 mRNA levels in acute myeloid leukaemia

To further study whether low-level expression of MeCP2 and MBD2 was restricted to carcinomas only or whether it was a general phenomenon in human malignancies, we analysed a panel of human acute myeloid leukaemias ($n$ = 24). Samples from 16 patients were obtained at the time of initial diagnosis and 8 samples were obtained from patients at the time of relapse. All leukaemic samples contained more than 70% blast cells. Normal bone marrow from patients in complete remission served as control ($n$ = 9). Expression of MeCP2 and MBD2 was higher in normal bone marrow than in

© 2001 Cancer Research Campaign

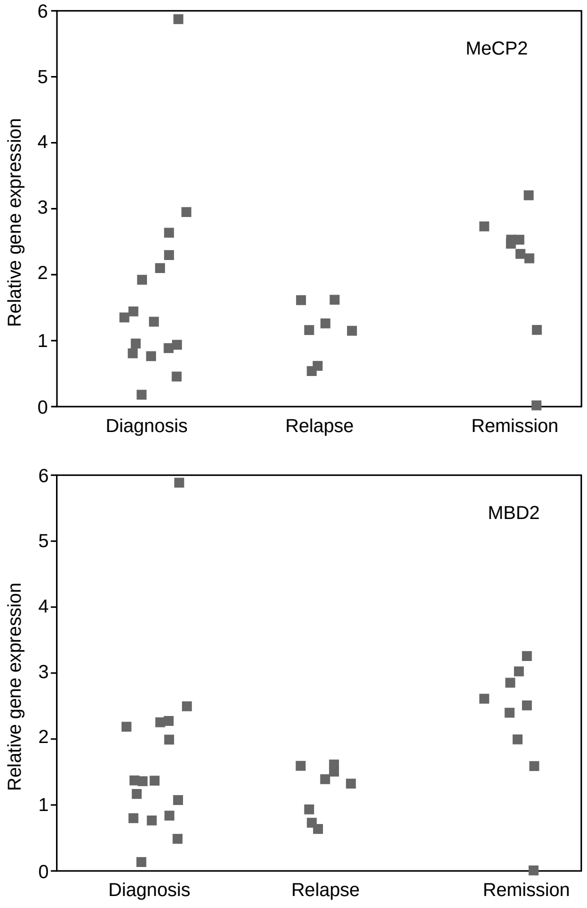

**Figure 3** Expression of MeCP2 and MBD2 in acute myeloid leukaemia. MeCP2 and MBD2 mRNA expression in blasts from patients with acute myeloid leukaemia at diagnosis ($n$ = 16) or at relapse ($n$ = 9) was compared to expression in bone marrow from patients in complete remission. Although a trend was seen towards lower expression levels in relapsed disease, no significant differences in expression were found

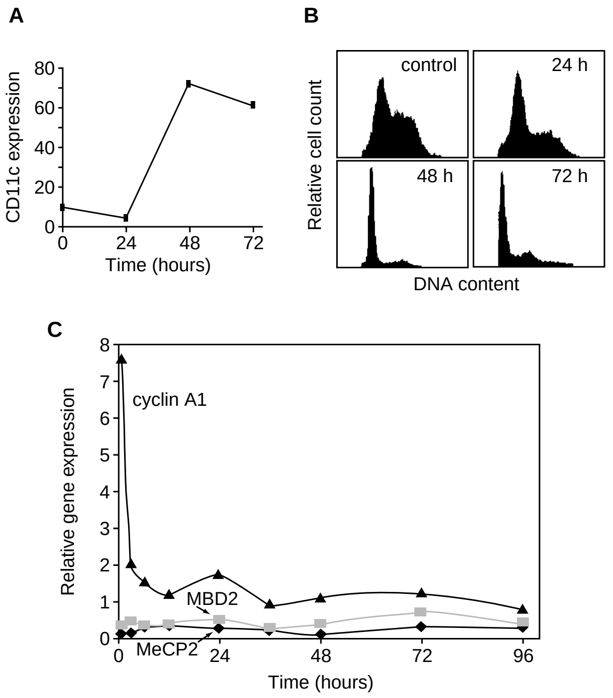

**Figure 4** Analyses of MBD expression during differentiation. (**A**) Differentiation of NB4 cells towards granulocytes was followed by flow cytometric staining for the surface antigen CD11c. (**B**) In addition, the cell cycle status was analysed at various time points to document the decrease in cycling cells that is associated with cellular differentiation. (**C**) While an increase in CD11c expression and a decrease of cells in S and G2/M phase were associated with a rapid decrease of cyclin A1 mRNA, no significant changes in MBD2 and MeCP2 expression levels were observed

AML blasts but this difference was statistically not significant (Figure 3). For patients with AML, MeCP2 and MBD2 expression levels were higher at diagnosis than at the time of relapse. However, most likely due to the small sample size, these differences were statistically not significant by one-way analyses of variance (MeCP2: $P$ = 0.24; MBD2: $P$ = 0.16). These data indicate that MeCP2 and MBD2 expression levels are probably somewhat lower in leukaemic blasts than in normal bone marrow but the differences appear to be much smaller than those detected in most carcinomas.

### MeCP2 and MBD2 are not cell cycle regulated and their expression is not associated with haematopoietic differentiation

Reduced gene expression in cancer might be linked to gene regulatory processes during the cell cycle or cellular differentiation. In vitro differentiation of leukaemic cells provides an easily accessible model to examine the roles of the cell cycle and differentiation mechanisms on gene expression levels. We used NB4 leukaemic cells that differentiated towards granulocytes upon exposure to ATRA and DMSO. Differentiation was controlled using morphology (not shown), differentiation surface marker expression and cell cycle analyses (Figure 4). In addition, we measured cyclin A1 mRNA as another control for granulocytic differentiation (Müller et al, 2000b). While cyclin A1 levels were rapidly down-regulated after NB4 cell exposure to ATRA and DMSO, no major changes were detected for MBD2 or MeCP2. Even when most cells were in G1 phase, expression levels of *MeCP2* and *MBD2* did not change, indicating that expression of these genes neither depended on the cell cycle nor on the state of cellular differentiation. Similar results were obtained, when U937 cells were differentiated towards monocytes upon TPA exposure (data not shown).

### DISCUSSION

Our study provides strong evidence for the loss of expression of MeCP2 and MBD2 in a substantial fraction of human cancers. This is the first time that expression of genes associated with CpG-methylation has been demonstrated to be consistently altered in human cancers. Expression of other methylation-associated genes such as DNA methyltransferase is currently controversially discussed (De Marzo et al, 1999; Eads et al, 1999; Xie et al, 1999).

We used quantitative real-time RT-PCR to analyse gene expression in a large panel of normal human organs as well as in multiple primary human tumours. We have previously demonstrated the feasibility of analysing *MBD* gene mRNA expression by real-time RT-PCR, and several precautions were taken to obtain reliable data (Müller et al, 2000a). First, all probes span an exon–exon junction, thus excluding the amplification of genomic DNA. Second, the results were consistent when different housekeeping genes were used for standardization purposes. The TATA-binding protein

*© 2001 Cancer Research Campaign*

(TBP) is expressed in the nucleus itself and has been shown to be a reliable housekeeping gene for standardization purposes (Bieche et al, 1999; Müller et al, 2000a). The *GAPDH* gene has been extensively used for this purpose as well. The data did not differ significantly whether the expression of the genes of the nuclear TBP or of the cytoplasmic GAPDH protein were used for standardization. Third, differences in gene expression levels could not be explained by differences in the cell cycle status. We showed that MBD2 and MeCP2 levels did not change upon exit of tumour cells from the cell cycle. Also, the NSCLC samples were additionally analysed for the proliferation markers PCNA and cyclin A2 (data not shown). The use of these genes for standardization purposes did not alter the detected loss of MBD2 and MeCP2 expression seen in the tumour samples.

To analyse whether reduced mRNA expression levels of MeCP2 corresponded to decreased protein expression as well, we performed Western blotting experiments. These experiments provided evidence that MeCP2 expression was reduced in NSCLC tumour cells on the protein level. High-quality antibodies to study expression of MBD2 at the protein level are currently not available.

The maintenance of CpG-methylation is essential for normal embryonic development and deletional mutants that disturb this process can be lethal (Li et al, 1992), e.g. the deletion of MeCP2 (Tate et al, 1996). MeCP2 was the first identified member of the Methyl-CpG-binding domain protein family and has been shown to be a transcriptional repressor. Mechanistically, MeCP2 acts by recruiting co-repressors and HDACs to methylated CpG dinucleotides (Nan et al, 1998). We and others have shown that human promoters can be transcriptionally repressed by MeCP2 when the promoter is methylated (Kudo, 1998; Müller et al, 2000a). MeCP2 has been shown to be mutated in RETT syndrome, a neurodevelopmental disorder (Amir et al, 1999). Point mutations occur in the majority of patients and these mutations diminish the protein's ability to bind to methylated DNA.

Since methylation has been shown to be associated with the silencing of tumour suppressor genes, *MeCP2* was considered to be an attractive candidate gene to mediate methylation-associated gene silencing in cancer. In the current study we provide strong evidence that MeCP2 mRNA expression is greatly reduced in a significant fraction of solid human cancers. This finding strongly argues against a role of MeCP2 in the silencing of methylated genes in cancer. It has already been hypothesized that MeCP2 expression levels might be too low in normal tissues to bind to all methylated DNA sequences (Nan et al, 1997). The reduced MeCP2 expression in human cancers suggests that MeCP2 function is disturbed in cancers.

MBD2 is another MBD family member that has been demonstrated to be associated with HDAC recruitment and subsequent transcriptional repression (Ng et al, 1999). No mutations of MBD2 have been detected in human disease so far, as have been for MeCP2. In contrast to the single protein encoded by the *MeCP2* gene, 2 isoforms of MBD2 occur and an additional testis-specific form is known (Hendrich and Bird, 1998; Hendrich et al, 1999). Potential functional differences between the MBD2 isoforms remain currently unknown. Therefore, we decided to use a primer and probe combination that could detect the 2 common isoforms but not the testis-specific transcript (Müller et al, 2000a). Our study showed that expression of *MBD2* gene is significantly reduced in a wide variety of primary human cancers. Similar findings have previously been reported for a small group of colorectal and gastric carcinomas (Kanai et al, 1999). The reasons for the loss of MBD2 expression and its functional consequences are unknown. However, the high frequency of this finding suggests a non-random mechanism. This is even more likely when one considers the high degree of correlation between loss of MBD2 and loss of MeCP2 expression in different cancers.

Another interesting point is that the loss of expression differed among cancer types. Our analyses showed the reduced expression to be especially pronounced in squamous cell carcinoma of the lung (data not shown). Also, expression in breast cancers appeared to be low while some other gynaecological malignancies such as cancer of the endometrium showed relatively high levels. In addition, only a minor reduction in MeCP2 and MBD2 expression was found in acute myeloid leukaemia.

More detailed analyses of *MBD2* and *MeCP2* gene expression were performed in non-small-cell lung cancer from patients in an early stage of disease. This homogenous patient population has been extensively characterized previously (Müller-Tidow et al, 2001). Interestingly, loss of expression of both genes occurred independently of the stage of the disease, histological grade or the sex of the patient. In addition, the reduction in neither MeCP2 nor MBD2 mRNA levels were associated with p53 mutational status and finally, expression levels of MeCP2 and MBD2 mRNA levels were not associated with the prognosis of the disease. These findings indicate that MeCP2 and MBD2 are likely to be down-regulated during early stages of the pathogenesis of most solid tumours. Down-regulation of these genes appears to occur in most tumours. Gross changes in genomic methylation patterns have been reported to be a common feature of human tumours as well (Costello et al, 2000). It is tempting to speculate that the loss of MBD protein expression and dysregulation of genomic methylation patterns are somehow connected.

Taken together, our study shows strong evidence for a loss of MeCP2 and MBD2 expression in multiple human solid tumours. The demonstrated relevance of the loss of MeCP2 function for human disease as well as the lethality of the MeCP2 knockout model suggest that the loss of MeCP2 expression might be relevant in the pathogenesis of cancer. The functional role of MBD2 as well as the reasons and consequences of its loss of expression in human cancers also need to be further studied.

### ACKNOWLEDGEMENTS

We are grateful to Annika Rudat for help with RNA preparation and cDNA synthesis. This work is part of the doctoral thesis of Katrin Kügler. This work is supported by grants from the Deutsche Krebshilfe (10-1539-Mü1), the Deutsche Forschungsgemeinschaft (Mu 1328/2-1 and 2-2, Se 600/2-2) and the IMF-Program (Mü429926, Mü529905, SE119908) at the University of Münster.

### REFERENCES

Amir RE, Van den Veyver IB, Wan M, Tran CQ, Francke U and Zoghbi HY (1999) RETT syndrome is caused by mutations in X-linked MeCP2, encoding methyl-CpG-binding protein 2. *Nat Genet* **23**: 185–188

Bieche I, Laurendeau I, Tozlu S, Olivi M, Vidaud D, Lidereau R and Vidaud M (1999) Quantitation of Myc gene expression in sporadic breast tumors with a real-time reverse transcription-PCR assay. *Cancer Res* **59**: 2759–2765

Bird AP (1996) The relationship of DNA methylation to cancer. *Cancer Surv* **28**: 87–101

Brandeis M, Ariel M and Cedar H (1993) Dynamics of DNA methylation during development. *Bioessays* **15**: 709–713

© 2001 Cancer Research Campaign

Chih DY, Chumakov AM, Park DJ, Silla AG and Koeffler HP (1997) Modulation of mRNA expression of a novel human myeloid-selective CCAAT/enhancer binding protein gene (C/EBP epsilon). *Blood* **90**: 2987–2994

Corn PG, Kuerbitz SJ, van Noesel MM, Esteller M, Compitello N, Baylin SB and Herman JG (1999) Transcriptional silencing of the p73 gene in acute lymphoblastic leukemia and Burkitt's lymphoma is associated with 5′ CpG island methylation. *Cancer Res* **59**: 3352–3356

Costello JF, Fruhwald MC, Smiraglia DJ, Rush LJ, Robertson GP, Gao X, Wright FA, Feramisco JD, Peltomaki P, Lang JC, Schuller DE, Yu L, Bloomfield CD, Caligiuri MA, Yates A, Nishikawa R, Su Huang H, Petrelli NJ, Zhang X, O'Dorisio MS, Held WA, Cavenee WK and Plass C (2000) Aberrant CpG-island methylation has non-random and tumour-type-specific patterns. *Nat Genet* **24**: 132–138

De Marzo AM, Marchi VL, Yang ES, Veeraswamy R, Lin X and Nelson WG (1999) Abnormal regulation of DNA methyltransferase expression during colorectal carcinogenesis. *Cancer Res* **59**: 3855–3860

Eads CA, Danenberg KD, Kawakami K, Saltz LB, Danenberg PV and Laird PW (1999) CpG island hypermethylation in human colorectal tumors is not associated with DNA methyltransferase overexpression. *Cancer Res* **59**: 2302–2306

Gaston K and Fried M (1995) CpG-methylation has differential effects on the binding of YY1 and ETS proteins to the bi-directional promoter of the Surf-1 and Surf-2 genes. *Nucleic Acids Res* **23**: 901–909

Heid CA, Stevens J, Livak KJ and Williams PM (1996) Real time quantitative PCR. *Genome Res* **6**: 986–994

Hendrich B and Bird A (1998) Identification and characterization of a family of mammalian methyl-CpG binding proteins. *Mol Cell Biol* **18**: 6538–6547

Hendrich B, Abbott C, McQueen H, Chambers D, Cross S and Bird A (1999) Genomic structure and chromosomal mapping of the murine and human Mbd1, Mbd2, Mbd3, and Mbd4 genes. *Mamm Genome* **10**: 906–912

Jones PL, Veenstra GJ, Wade PA, Vermaak D, Kass SU, Landsberger N, Strouboulis J and Wolffe AP (1998) Methylated DNA and MeCP2 recruit histone deacetylase to repress transcription. *Nat Genet* **19**: 187–191

Kanai Y, Ushijima S, Nakanishi Y and Hirohashi S (1999) Reduced mRNA expression of the DNA demethylase, MBD2, in human colorectal and stomach cancers. *Biochem Biophys Res Commun* **264**: 962–966

Kawano S, Miller CW, Gombart AF, Bartram CR, Matsuo Y, Asou H, Sakashita A, Said J, Tatsumi E and Koeffler HP (1999) Loss of p73 gene expression in leukemias/lymphomas due to hypermethylation. *Blood* **94**: 1113–1120

Kudo S (1998) Methyl-CpG-binding protein MeCP2 represses Sp1-activated transcription of the human leukosialin gene when the promoter is methylated. *Mol Cell Biol* **18**: 5492–5499

Laird PW and Jaenisch R (1996) The role of DNA methylation in cancer genetic and epigenetics. *Annu Rev Genet* **30**: 441–464

Li E, Bestor TH and Jaenisch R (1992) Targeted Mutation of the DNA methyltransferase gene results in embryonic lethality. *Cell* **69**: 915–926

Liang G, Salem CE, Yu MC, Nguyen HD, Gonzales FA, Nguyen TT, Nichols PW and Jones PA (1998) DNA methylation differences associated with tumor tissues identified by genome scanning analysis. *Genomics* **53**: 260–268

Müller-Tidow C, Metzger R, Kügler K, Diederichs S, Thomas M, Schneider P, Koeffler HP, Berdel WE and Serve H (2001) Cyclin E is the only CDK2 associated Cyclin that predicts metastasis and survival in early stage non-small cell lung cancer. *Cancer Res* in press.

Müller C, Readhead C, Diederichs S, Idos G, Yang R, Tidow N, Serve H, Berdel WE and Koeffler HP (2000a) Methylation of the cyclin A1 promoter correlates with gene silencing in somatic cell lines, while tissue-specific expression of cyclin A1 is methylation independent. *Mol Cell Biol* **20**: 3316–3329

Müller C, Yang R, Park DJ, Serve H, Berdel WE and Koeffler HP (2000b) The aberrant fusion proteins PML-RARa and PLZF-RARa contribute to the overexpression of Cyclin A1 in acute promyelocytic leukemia. *Blood* **96**: 3894–3899

Nakamura M, Sugita K, Inukai T, Goi K, Iijima K, Tezuka T, Kojika S, Shiraishi K, Miyamoto N, Karakida N, Kagami K, T OK, Mori T and Nakazawa S (1999) p16/MTS1/INK4A gene is frequently inactivated by hypermethylation in childhood acute lymphoblastic leukemia with 11q23 translocation. *Leukemia* **13**: 884–890

Nan X, Campoy FJ and Bird A (1997) MeCP2 is a transcriptional repressor with abundant binding sites in genomic chromatin. *Cell* **88**: 471–481

Nan X, Ng HH, Johnson CA, Laherty CD, Turner BM, Eisenman RN and Bird A (1998) Transcriptional repression by the methyl-CpG-binding protein MeCP2 involves a histone deacetylase complex. *Nature* **393**: 386–389

Ng HH, Zhang Y, Hendrich B, Johnson CA, Turner BM, Erdjument-Bromage H, Tempst P, Reinberg D and Bird A (1999) MBD2 is a transcriptional repressor belonging to the MeCP1 histone deacetylase complex. *Nat Genet* **23**: 58–61

Ng HH, Jeppesen P and Bird A (2000) Active repression of methylated genes by the chromosomal protein MBD1. *Mol Cell Biol* **20**: 1394–1406

Pradhan S, Talbot D, Sha M, Benner J, Hornstra L, Li E, Jaenisch R and Roberts RJ (1997) Baculovirus-mediated expression and characterization of the full length murine DNA methyltranferase. *Nucleic Acid Res* **25**: 4666–4673

Santoli D, Francis MK, Matera L and Ferrero D (1983) Induction of proliferation and NK activity in human lymphocytes by mature myelomonocytic cells. *J Immunol* **131**: 736–742

Shapiro GI, Park JE, Edwards CD, Mao L, Merlo A, Sidransky D, Ewen ME and Rollins BJ (1995) Multiple mechanisms of p16INK4A inactivation in non-small cell lung cancer cell lines. *Cancer Res* **55**: 6200–6209

Singal R and Ginder GD (1999) DNA Methylation. *Blood* **93**: 4059–4070

Song SH, Jong HS, Choi HH, Kang SH, Ryu MH, Kim NK, Kim WH and Bang YJ (2000) Methylation of specific CpG sites in the promoter region could significantly down-regulate p16(INK4a) expression in gastric adenocarcinoma. *Int J Cancer* **87**: 236–240

Swafford DS, Middleton SK, Palmisano WA, Nikula KJ, Tesfaigzi J, Baylin SB, Herman JG and Belinsky SA (1997) Frequent aberrant methylation of p16INK4a in primary rat lung tumors. *Mol Cell Biol* **17**: 1366–1374

Tate P, Skarnes W and Bird A (1996) The methyl-CpG binding protein MeCP2 is essential for embryonic development in the mouse. *Nat Genet* **12**: 205–208

Walsh CP and Bestor TH (1999) Cytosine Methylation and mammalian development. *Genes Dev* **13**: 26–34

Warnecke PM and Clark SJ (1999) DNA methylation profile of the mouse skeletal alpha-actin promoter during development and differentiation. *Mol Cell Biol* **19**: 164–172

Xie S, Wang Z, Okano M, Nogami M, Li Y, He WW, Okumura K and Li E (1999) Cloning, expression and chromosome locations of the human DNMT3 gene family. *Gene* **236**: 87–95

*© 2001 Cancer Research Campaign*
